# Personality Trait Dimensions and Depressive Symptoms Among Medical Students: Sequential Mediation Through Psychological Capital and Perceived Stress

**DOI:** 10.1155/da/9727081

**Published:** 2026-08-06

**Authors:** Ziqi Wang, Simeng Wang, Deliang Wen

**Affiliations:** ^1^ Institute of Health Professions Education Assessment and Reform, China Medical University, Shenyang 110122, Liaoning Province, China, cmu.edu.cn; ^2^ Health Sciences Institute, China Medical University, Shenyang 110122, Liaoning Province, China, cmu.edu.cn

**Keywords:** depressive symptoms, medical students, perceived stress, personality traits, psychological capital

## Abstract

**Background:**

Depressive symptoms (DSs) are highly prevalent among medical students and may adversely affect both student well‐being and future healthcare quality. Although personality traits (PTs) are closely associated with mental health, the psychological processes underlying these associations remain incompletely understood. This study examined the associations between the five PT dimensions and DS and explored whether psychological capital (PsyCap) and perceived stress (PS) statistically mediated these associations.

**Methods:**

A multicenter cross‐sectional survey was conducted among 583 third‐year undergraduate clinical medicine students from three medical universities in China. Standardized questionnaires were administered to assess PTs, PsyCap, PS, and DS. Correlation, regression, and sequential mediation analyses were performed.

**Results:**

Correlation analyses showed that neuroticism was negatively correlated with PsyCap and positively correlated with PS and DS, whereas extraversion, openness to experience, agreeableness, and conscientiousness demonstrated the opposite pattern (all *p* < 0.001). Regression analyses indicated that neuroticism, openness to experience, agreeableness, and conscientiousness were significantly associated with DS (*β* = 0.39, −0.14, −0.15, −0.10, respectively, all *p* < 0.001), whereas the direct association between extraversion and DS was not statistically significant (*β* = −0.05). Mediation analyses supported statistically significant indirect associations through PsyCap alone, PS alone, and the proposed sequential pathway through PsyCap and PS. Among the five PT dimensions, the association between extraversion and DS was primarily reflected through the indirect pathways.

**Conclusion:**

The findings suggest that PTs may be associated with DS through psychological resources and stress appraisal. By integrating PsyCap and PS into a sequential mediation framework, this study provides an expanded perspective on the psychological processes linking PTs and DS among medical students. Given the cross‐sectional design, the proposed mediation pathways should be interpreted as statistical associations rather than causal relationships. Future longitudinal and intervention studies are needed to further evaluate these associations.

## 1. Introduction

Depression has emerged as a significant public health issue among university students worldwide, particularly those enrolled in medical programs [1, 2]. Medical students are frequently exposed to intense academic workloads, highly competitive environments, long study hours, and emotionally demanding clinical experiences, which together create a particularly high‐risk context for the development of psychological distress [3–7]. Epidemiological evidence indicates that depressive symptoms (DSs) affect approximately 20%–50% of medical students, a prevalence consistently higher than that reported in the general university student population [8–10]. In China, recent surveys have similarly reported high levels of DS among undergraduate medical students, with a considerable proportion experiencing moderate to severe psychological difficulties [11, 12]. Together, these findings underscore the need not only to better understand the determinants of DS among medical students but also to identify psychological mechanisms that may distinguish this population from other university students.

The consequences of depression in medical students are multifaceted and far‐reaching. Persistent DS can impair cognitive functioning [13], reduce motivation, and hinder learning [6, 14], which in turn compromises academic achievement [15]. It is also strongly associated with burnout [16], dropout intention [17], substance use [18], and suicidal ideation [19, 20]. These adverse outcomes extend beyond the individual, potentially undermining the quality of care delivered during clinical training and later professional practice [21, 22]. Therefore, promoting mental well‐being among medical students is essential not only for their personal and professional development but also for safeguarding the quality, safety, and sustainability of future healthcare systems from both public health and medical education perspectives.

Given the high prevalence and adverse consequences of DS among medical students, researchers have increasingly focused on identifying psychological factors that may exacerbate or buffer the risk of depression in this group [23–25]. Personality traits (PTs) represent one such factor. Across diverse populations, empirical evidence consistently suggests that high neuroticism is strongly associated with DS, whereas conscientiousness, extraversion, openness to experience, and agreeableness are generally considered protective factors [26–29]. However, whether these associations are equally applicable to medical students remains insufficiently understood, as this population is exposed to unique academic, clinical, and professional demands that may alter the relationships between personality and mental health outcomes. Therefore, clarifying whether these personality‐depression associations are similarly observed among medical students is important for identifying individuals at elevated risk and developing targeted prevention and intervention strategies tailored to this population.

Psychological capital (PsyCap) and perceived stress (PS) have both been identified as important psychological correlates of DS among medical students [30–33]. Individuals with higher levels of PsyCap generally demonstrate greater optimism, resilience, hope, and self‐efficacy, enabling them to cope more effectively with academic and emotional challenges [33, 34]. Consistent evidence has shown that higher PsyCap is associated with lower levels of DS across diverse populations, including university students and healthcare professionals [35, 36]. Likewise, although objective stressors contribute to psychological distress, PS (i.e., individuals’ subjective appraisal of stressful experiences) has consistently been shown to be a stronger predictor of mental health outcomes than objective stress exposure [37].

From a theoretical perspective, PsyCap may represent an important psychological resource that influences how individuals appraise stressful situations. According to the Broaden‐and‐build theory of positive emotions and the transactional model of stress and coping, positive psychological resources may promote broader cognitive and behavioral responses to challenges and facilitate more adaptive stress appraisals, thereby reducing PS [38]. Consistent with this perspective, certain PTs, particularly neuroticism, have been associated with elevated PS, whereas higher PsyCap may buffer these perceptions by promoting more adaptive coping strategies [39]. Although prolonged PS may also undermine psychological resources over time [40], the present study follows the theoretically derived pathway in which PsyCap precedes PS, while acknowledging that alternative temporal sequences should be examined in future longitudinal research. Although previous studies have separately linked PTs, PsyCap, and PS to DS, few studies have examined whether PsyCap and PS jointly and sequentially explain the associations between the five PT dimensions and DS among medical students. Therefore, this study aimed to examine the associations between the five PT dimensions and DS and to investigate whether PsyCap and PS statistically mediate these associations in a sequential manner.

## 2. Theoretical Framework and Hypotheses

The predisposition model conceptualizes PTs as relatively stable predispositions that shape individuals’ vulnerability to depression by influencing how they perceive, interpret, and respond to environmental demands [41, 42]. Consistent with this perspective, the five‐factor model (FFM) describes personality using five broad dimensions: neuroticism, extraversion, openness to experience, agreeableness, and conscientiousness [43, 44]. These traits are thought to influence emotional regulation, cognitive appraisal, coping strategies, and interpersonal functioning, thereby contributing to individual differences in mental health outcomes [45–50]. Among the five dimensions, neuroticism has consistently been identified as the strongest risk factor for DS because individuals high in neuroticism tend to experience greater emotional instability, heightened sensitivity to negative events, and maladaptive cognitive patterns such as rumination and catastrophizing [45, 46]. By contrast, extraversion, openness to experience, agreeableness, and conscientiousness are generally considered protective characteristics that facilitate adaptive coping, emotional regulation, and supportive social relationships [51, 52]. Although the strength of these associations varies across populations, substantial empirical evidence has demonstrated that higher neuroticism is associated with greater DS, whereas the remaining personality dimensions are generally associated with lower DS [53–57]. Although these associations have been well documented in adolescents and the general population, relatively fewer studies have examined these relationships specifically among medical students [58]. Medical students experience unique educational and clinical demands that may influence how personality characteristics are associated with DS. Therefore, examining these associations within medical student populations may help determine whether previously established personality–depression relationships are generalizable to this population and facilitate the early identification of students at elevated risk for depression. Based on the above theoretical and empirical evidence, we proposed the Hypothesis 1: Each of the five PT dimensions will significantly predict DS among medical students (H1a–H1e).

In addition to relatively stable personality dispositions, modifiable psychological resources have increasingly been recognized as important determinants of mental health [59, 60]. PsyCap is a higher‐order positive psychological construct comprising four core components: hope, self‐efficacy, resilience, and optimism [61, 62]. According to positive psychology and the Conservation of Resources (COR) theory, these psychological resources enable individuals to maintain motivation, adopt adaptive coping strategies, and recover more effectively from adversity, thereby promoting psychological well‐being and protecting against emotional distress [38, 63]. PTs are considered important antecedents of PsyCap. Previous studies have consistently reported meaningful associations between the five major PTs and PsyCap [64, 65]. Specifically, neuroticism is generally negatively associated with hope, self‐efficacy, resilience, and optimism, whereas extraversion, openness to experience, agreeableness, and conscientiousness are positively associated with these components [64, 66, 67]. Individuals with these adaptive PTs are therefore more likely to accumulate positive psychological resources, whereas those high in neuroticism may be less likely to develop such resources because of persistent negative affect and maladaptive cognitive tendencies [68]. A growing body of evidence further suggests that higher PsyCap is associated with lower levels of anxiety and DS, greater well‐being, and more effective coping with academic and life stressors among university students and healthcare professionals [62, 69–75]. Moreover, PsyCap has been shown to mediate the relationships between various psychosocial factors, including life satisfaction, physical activity, and interpersonal sensitivity, and DS in both college and medical student populations [60, 76, 77]. Collectively, these findings suggest that PsyCap may represent an important psychological pathway through which PTs are associated with DS. Based on the above theoretical and empirical evidence, we proposed the Hypotheses 2–4: Each of the five PT dimensions will significantly predict PsyCap among medical students (H2a–H2e). PsyCap will be negatively associated with DS (H3). PsyCap will statistically mediate the associations between each of the five PT dimensions and DS (H4a–H4e).

PS is another important psychological correlate of DS, referring to individuals’ subjective appraisal of the extent to which environmental demands exceed their perceived coping capacity [78]. According to the Cognitive Appraisal Theory of Stress, psychological responses to stressful events are determined not only by objective stressors but also by individuals’ cognitive evaluations of those stressors and their perceived ability to cope with them [79]. Consequently, PS captures important information beyond objective stress exposure by reflecting both subjective stress appraisal and perceived coping resources [80]. PTs are considered important determinants of how individuals perceive and respond to stressful situations [81–83]. Consistent evidence indicates that individuals with higher neuroticism tend to experience greater negative affect and higher levels of PS, whereas extraversion, openness to experience, and agreeableness are generally associated with lower levels of PS [84–87]. Findings regarding conscientiousness are somewhat inconsistent, with some studies reporting positive associations with PS and others suggesting that conscientious individuals are more likely to adopt adaptive coping strategies, such as problem‐focused coping, thereby reducing PS [88, 89]. Collectively, these findings suggest that individual differences in PS may partly reflect underlying PTs. Furthermore, higher levels of PS have consistently been associated with greater DS across diverse populations [24, 90, 91]. Previous studies have suggested that PS statistically mediates the relationships between PTs and psychological outcomes. For example, the structural model proposed by Roohafza et al. [92] highlighted PS as an important pathway linking PTs to psychological health, and a large population‐based study involving 3950 Korean adults further demonstrated that PS mediated the association between PTs and DS [87]. Together, these theoretical and empirical findings support the proposition that PS may represent an important psychological mechanism through which PTs are associated with DS. Based on the above theoretical and empirical evidence, we proposed the Hypotheses 5–7: Each of the five PT dimensions will significantly predict PS among medical students (H5a–H5e). PS will be positively associated with DS (H6). PS will statistically mediate the associations between each of the five PT dimensions and DS (H7a–H7e).

**Figure 1:**
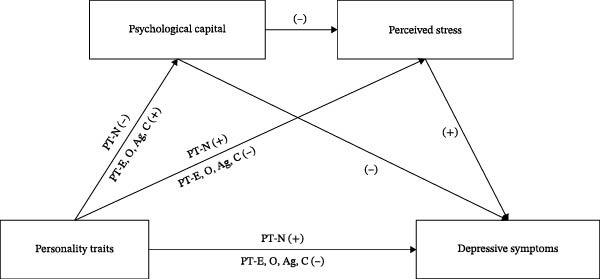
Diagram of the hypothetical model. Abbreviations: Ag, agreeableness; C, conscientiousness; E, extraversion; N, neuroticism; O, openness to experience; PT, personality trait.

Although PsyCap and PS have each been examined as mediators of mental health outcomes, relatively little research has investigated their combined or sequential mediating roles, particularly among medical students. Existing evidence consistently demonstrates a negative association between PsyCap and PS [88–91]. However, the theoretical basis underlying the temporal ordering of these two constructs warrants further clarification. According to the Broaden‐and‐build theory of positive emotions and the COR theory, PsyCap represents an internal psychological resource that facilitates adaptive cognitive appraisal and coping [61]. Individuals with greater hope, optimism, resilience, and self‐efficacy are more likely to perceive challenging situations as manageable rather than overwhelming, thereby experiencing lower levels of PS [93]. From this perspective, PsyCap is theoretically positioned as an antecedent of PS because positive psychological resources are expected to shape how individuals interpret and respond to environmental demands. Nevertheless, it is also plausible that prolonged exposure to high levels of PS may gradually deplete psychological resources over time, suggesting that reciprocal or alternative temporal relationships may exist [40]. Because the present study employed a cross‐sectional design, these competing temporal sequences cannot be empirically distinguished. Therefore, the proposed “PsyCap → PS” pathway should be regarded as a theory‐driven statistical mediation model, rather than evidence of temporal or causal ordering. Building upon the theoretical and empirical evidence presented above, we propose that PTs may be associated with DS through a sequential pathway in which personality influences PsyCap, PsyCap is associated with lower PS, and lower PS is associated with fewer DS. Clarifying this sequential pathway may contribute to a more comprehensive understanding of the psychological processes linking PTs and DS among medical students. Based on the above theoretical and empirical evidence, we proposed the Hypotheses 8 and 9: PsyCap will be negatively associated with PS (H8). PsyCap and PS will statistically exert a sequential mediating role in the associations between each of the five PT dimensions and DS. The conceptual framework of the proposed hypotheses is presented in Figure 1.

## 3. Methods

### 3.1. Study Design and Participants

This study employed a multicenter cross‐sectional design. Data were collected between November 2021 and January 2022, during the coronavirus disease 2019 (COVID‐19) pandemic, from three public medical institutions in Liaoning Province (China Medical University, Dalian Medical University, and Shenyang Medical College), China. These institutions were selected to enhance sample representativeness within Liaoning Province because they differ in the institutional level, geographical location, and educational characteristics. Nevertheless, because all participating institutions were located within a single province, caution is warranted when generalizing the findings to medical students in other regions of China.

The study focused on third‐year undergraduate students majoring in clinical medicine. This group was selected because third‐year students had generally completed the transition from preclinical to discipline‐specific medical education while not yet entering the internship and employment stages, thereby reducing heterogeneity attributable to different phases of medical training. In addition, previous studies have suggested that the prevalence of DS among third‐year medical students is intermediate relative to other academic years, making this population particularly suitable for examining psychological factors associated with DS [11, 94, 95]. Eligible participants were full‐time third‐year undergraduate students majoring in clinical medicine who voluntarily provided informed consent. Students with a self‐reported diagnosis of schizophrenia or those currently on academic suspension were excluded.

The required sample size was estimated using the single population proportion formula (Formula (1)) [96]. Assuming a prevalence of DS of approximately 30% among medical students [11], a 95% confidence interval (CI), and a 5% margin of error (MOE), the minimum required sample size was calculated to be 323 participants. To account for an anticipated invalid response rate of approximately 20%, the target sample size was increased to 388 participants.

(1) N=Zα/22×P×1−PMOE2.

### 3.2. Procedure

After obtaining approval from the participating universities, data collection was conducted during scheduled class sessions. The researchers introduced the study objectives and procedures, emphasizing that participation was voluntary, responses would remain anonymous, and participation or nonparticipation would not affect students’ academic performance. The survey consisted exclusively of standardized self‐report questionnaires and did not involve the collection of sensitive personal information. Before participation, all students were asked to read and sign an informed consent form. Only students who provided written informed consent were eligible to complete the anonymous questionnaire. The electronic questionnaire was administered through the Wenjuanxing online survey platform (https://www.wjx.cn/), which participants accessed by scanning a QR code using WeChat.

To ensure the quality and integrity of the data, several quality‐control procedures were implemented. First, IP addresses and access permissions were restricted to prevent duplicate submissions, with each participant permitted to complete the questionnaire only once. Second, questionnaires completed in less than 3 min, as automatically recorded by the Wenjuanxing platform, were excluded. Third, questionnaires exhibiting straight‐line response patterns or obvious logical inconsistencies were removed during data screening. In addition, participants received a small incentive after completing the questionnaire to encourage participation.

Based on the distribution of third‐year undergraduate clinical medicine students across the three participating institutions, 616 students from 20 randomly selected classes were invited to participate. Classes were selected using cluster random sampling within each participating university. A total of 603 questionnaires were returned. After excluding 20 questionnaires because of excessively short completion times, straight‐line response patterns, or obvious logical inconsistencies, 583 valid questionnaires were retained for the final analyses, yielding an effective response rate of 94.6%. The final sample substantially exceeded the minimum sample size required for this study.

### 3.3. Measures

The outcome variable was DS, the five PT dimensions served as the independent variables, PsyCap, and PS were specified as sequential mediators, and demographic characteristics were included as covariates.

#### 3.3.1. DSs

DSs were assessed using the validated Chinese version of the 20‐item Center for Epidemiologic Studies Depression Scale (CES‐D) [97]. The CES‐D measures the frequency of DS experienced during the previous week and consists of 16 negatively worded items and four positively worded items, with the latter reverse‐scored before calculating the total score. Each item was rated on a four‐point Likert scale ranging from 0 (“rarely or none of the time”) to 3 (“most or all of the time”), yielding total scores ranging from 0 to 60, with higher scores indicating more severe DS. The Chinese version of the CES‐D has been extensively validated and widely used in Chinese populations, including university students and adolescents [98, 99]. Although a cutoff score of 16 has traditionally been used to identify individuals at risk of depression [100], subsequent validation studies have shown that the optimal threshold varies across populations [101–103]. A systematic review and meta‐analysis of 28 validation studies suggested that a cutoff score of 20 provides improved diagnostic accuracy for identifying elevated DS in the general population [102]. Moreover, subsequent studies have demonstrated that this cutoff is also appropriate for Chinese university students [98]. Therefore, consistent with previous validation studies, a cutoff score of 20 was adopted in the present study to identify students with elevated DS.

#### 3.3.2. PTs

PTs were assessed using the Chinese version of the 60‐item Neuroticism–Extraversion–Openness Five‐Factor Inventory‐Revised (NEO‐FFI‐R), originally developed by McCrae and Costa and subsequently revised for use in Chinese populations [104, 105]. The NEO‐FFI‐R comprises 60 items equally distributed across five personality dimensions: neuroticism, extraversion, openness to experience, agreeableness, and conscientiousness, with each subscale containing 12 items. Responses were rated on a 5‐point Likert scale ranging from 1 (“strongly disagree”) to 5 (“strongly agree”), with higher scores indicating higher levels of the corresponding PT. The Chinese version of the NEO‐FFI‐R has demonstrated satisfactory reliability and validity among Chinese university students and adolescent populations [106, 107].

#### 3.3.3. PsyCap

PsyCap was assessed using the Positive PsyCap Questionnaire (PPQ), which was developed by Zhang et al. [108] for use in Chinese populations based on the PPQ proposed by Luthans et al. [109]. The PPQ consists of 26 items encompassing four core dimensions: self‐efficacy, resilience, hope, and optimism. All items were rated on a 7‐point Likert scale ranging from 1 (“strongly disagree”) to 7 (“strongly agree”), with higher total scores indicating higher levels of PsyCap. The PPQ has demonstrated satisfactory reliability and validity among Chinese medical student populations [59, 110, 111]. Consistent with the conceptualization of PsyCap as a higher‐order psychological construct, only the overall PPQ score was used in the present study to represent participants’ overall level of PsyCap.

#### 3.3.4. PS

PS was assessed using the validated Chinese version of the 10‐item PS Scale (PSS‐10) [112]. PSS‐10 assesses the degree to which individuals perceive their lives as stressful during the previous month. Each item is rated on a 5‐point Likert scale ranging from 0 (“never”) to 4 (”often”), with higher total scores indicating higher levels of PS [113, 114]. The Chinese version of the PSS‐10 has been widely used among Chinese university students, including medical student populations, and has demonstrated satisfactory reliability and validity [115–118].

#### 3.3.5. Covariates

Demographic variables included gender, place of residence, only‐child status, parental educational attainment, and regular exercise. Because all participants were third‐year undergraduate students majoring in clinical medicine, age, academic year, and major showed minimal variability and were therefore not included as covariates in the analyses.

### 3.4. Data Analysis

All statistical analyses were performed using IBM SPSS Statistics version 26.0 (IBM Corp., Armonk, NY, USA). A two‐sided *p* value <0.05 was considered statistically significant. Potential common method bias (CMB) was evaluated using Harman’s single‐factor test. The psychometric properties of all measurement instruments were assessed by examining internal consistency, composite reliability (CR), structural validity, and convergent validity. Descriptive statistics, including means, standard deviations (SDs), frequencies, and percentages, were calculated to summarize participants’ characteristics. Independent‐samples *t* tests and one‐way analyses of variance (ANOVA) were used to compare DS across demographic subgroups. Before correlation analyses, the normality of continuous variables (PTs, PsyCap, PS, and DS) was assessed. Pearson’s correlation coefficients were calculated for normally distributed variables, whereas Spearman’s rank correlation coefficients were used when the assumption of normality was violated. Multiple regression analyses and Model 6 of the PROCESS macro (version 4.2) were used to estimate the proposed sequential mediation models. Each of the five PT dimensions was analyzed separately as an independent variable, DS as the dependent variable, and PsyCap and PS as sequential mediators in the mediation analyses. Demographic variables were entered as covariates in all mediation analyses. Multicollinearity was evaluated using the variance inflation factor (VIF), with values below 5 indicating no evidence of problematic multicollinearity. Indirect effects were estimated using 5000 bias‐corrected bootstrap resamples. An indirect effect was considered statistically significant when the 95% bias‐corrected CI did not include zero. Given the cross‐sectional design of this study, the mediation analyses were interpreted as tests of theory‐driven statistical associations rather than evidence of temporal or causal relationships.

### 3.5. Ethics

The study protocol was reviewed and approved by the Ethics Committee of China Medical University (Approval No. 2019079) and was conducted in accordance with the principles of the Declaration of Helsinki. Before data collection, all participants received detailed information about the study objectives, procedures, potential risks, and their rights as research participants, and informed consent was obtained from each participant. Participation was entirely voluntary, and participants were free to withdraw from the study at any time without penalty. No personally identifiable information was collected. All data were anonymized, used solely for research purposes, and stored under strict confidentiality procedures.

## 4. Results

### 4.1. CMB

To minimize the CMB, several procedural remedies were implemented, including anonymous data collection, randomization of item order, and reverse scoring of selected items. Because all variables were assessed using self‐report questionnaires collected from a single source, CMB remained a potential concern. Potential CMB was examined using Harman’s single‐factor test based on exploratory factor analysis (EFA) [119]. EFA was conducted using all measurement items representing the four study constructs (the five PT dimensions, PsyCap, PS, and DS). The unrotated factor solution yielded 17 factors with eigenvalues greater than 1, and the first unrotated factor accounted for 25.98% of the total variance, which was below the commonly accepted threshold of 40% [120]. These findings indicate that no substantial CMB was detected in the present study.

### 4.2. Measurement Properties

The reliability and validity of all measurement instruments were evaluated prior to hypothesis testing. Internal consistency was assessed using Cronbach’s α, with all coefficients exceeding 0.70, indicating satisfactory internal consistency. Sampling adequacy was supported by Kaiser–Meyer–Olkin (KMO) values greater than 0.70, and the total variance explained by each measurement scale exceeded 50%, supporting the suitability of the data for factor analysis [121]. All CR coefficients exceeded 0.90, indicating satisfactory construct reliability. In addition, average variance extracted (AVE) was used to evaluate convergent validity. According to the recommended criteria [122, 123], all AVE values met the acceptable thresholds. Detailed results are presented in Table 1.

**Table 1:** Assessment of measurement model (*N* = 583).

Measures	Cronbach’s α	KMO	TVE (%)	CR	AVE
NEO‐FFI‐R	0.944	0.947	61.7	0.977	0.424
NEO‐FFI‐R‐N	0.781	0.878	56.7	0.938	0.562
NEO‐FFI‐R‐E	0.789	0.850	65.6	0.936	0.553
NEO‐FFI‐R‐O	0.757	0.823	53.8	0.928	0.522
NEO‐FFI‐R‐Ag	0.769	0.796	56.3	0.915	0.478
NEO‐FFI‐R‐C	0.823	0.901	65.0	0.956	0.646
PPQ	0.939	0.958	68.8	0.971	0.568
PSS	0.825	0.869	74.2	0.965	0.736
CES‐D	0.909	0.955	66.6	0.973	0.647

Abbreviations: Ag, agreeableness; AVE, average variance extracted; C, conscientiousness; CES‐D, the center for epidemiologic studies depression scale; CR, composite reliability; E, extraversion; KMO, Kaiser‐Meyer‐Olkin value; N, neuroticism; NEO‐FFI‐R, the neuroticism–extraversion–openness five‐factor inventory‐revised; O, openness; PPQ, the positive psychological capital questionnaire; PSS, the perceived stress scale; TVE, total variance explained.

### 4.3. Participant Characteristics

As shown in Table 2, the final sample comprised 583 medical students, including 260 (44.6%) men and 323 (55.4%) women, with approximately 60% of the participants residing in urban areas. The most common paternal educational level was a college degree or above (42.7%), whereas the most common maternal educational level was junior middle school or below (42.2%). Based on the predefined CES‐D cutoff score of ≥20, 60.9% of participants were classified as having elevated DS. Independent‐samples *t* tests showed that male students reported significantly higher levels of DS than female students (*p* < 0.05). No statistically significant differences in DS were observed across the remaining demographic characteristics (*p* > 0.05).

**Table 2:** Comparison of the score for depressive symptoms by sample characteristics (*N* = 583).

Variables	*N* (%)	Depressive symptoms (mean ± SD)	*t/F* ^a^	*p*
Gender				
Male	260 (44.6%)	21.86 ± 11.22	2.347	0.019^∗^
Female	323 (55.4%)	19.72 ± 10.69		
Place of residence				
Urban areas	345 (59.2%)	20.15 ± 11.68	−1.433	0.152
Rural areas	238 (40.8%)	21.44 ± 9.84		
Only‐child				
Yes	325 (55.7%)	20.78 ± 11.32	0.249	0.803
No	258 (44.3%)	20.55 ± 10.53		
Father’s education level				
Junior middle school or below	226 (38.8%)	21.54 ± 9.85	1.159	0.315
Senior middle school	108 (18.5%)	20.23 ± 10.61		
College degree or above	249 (42.7%)	20.08 ± 12.04		
Mother’s education level				
Junior middle school and below	246 (42.2%)	21.17 ± 9.72	0.623	0.537
Senior middle school	114 (19.6%)	20.85 ± 10.72		
College degree and above	223 (38.3%)	20.05 ± 12.34		
Exercise				
Yes	536 (91.9%)	20.79 ± 11.01	0.848	0.400
No	47 (8.1%)	19.43 ± 10.52		

^a^Independent sample *t* test and one‐way analyses of variance were used.

^∗^ *p* < 0.05.

### 4.4. Correlation Analysis

The normality of all continuous variables was assessed using the one‐sample Kolmogorov–Smirnov (K–S) test. Descriptive statistics, normality indices, and the correlation matrix are presented in Table 3. The K–S test indicated that all continuous variables deviated significantly from normality; therefore, Spearman’s rank correlation analyses were conducted. As shown in Table 3, agreeableness showed a comparatively weaker positive correlation with PsyCap (*ρ* = 0.245, *p* < 0.001), whereas the remaining PT dimensions demonstrated moderate to strong correlations with PsyCap, PS, and DS (|*ρ*| = 0.441–0.746, all *p* < 0.001). In addition, PsyCap was negatively correlated with both PS and DS, whereas PS was positively correlated with DS (all *p* < 0.001).

**Table 3:** Descriptive statistics, normality parameters, and correlation matrices between five personality traits dimensions, psychological capital, perceived stress, and depressive symptoms (*N* = 583).

Variables	Mean ± SD	NP	PT‐N	PT‐E	PT‐O	PT‐Ag	PT‐C	PsyCap	PS	DS
PT‐N	32.90 ± 7.26	122.62 ± 23.74^∗∗^	1	−0.438^∗∗^	−0.504^∗∗^	−0.521^∗∗^	−0.501^∗∗^	−0.506^∗∗^	0.743^∗∗^	0.746^∗∗^
PT‐E	38.61 ± 6.18	20.68 ± 10.97^∗∗^	—	1	0.413^∗∗^	0.134^∗^	0.606^∗∗^	0.585^∗∗^	−0.442^∗∗^	−0.467^∗∗^
PT‐O	40.14 ± 5.43	26.96 ± 6.25^∗∗^	—	—	1	0.430^∗∗^	0.635^∗∗^	0.482^∗∗^	−0.455^∗∗^	−0.519^∗∗^
PT‐Ag	38.74 ± 5.06	32.90 ± 7.26^∗∗^	—	—	—	1	0.326^∗∗^	0.245^∗∗^	−0.441^∗∗^	−0.470^∗∗^
PT‐C	41.27 ± 6.72	38.61 ± 6.18^∗∗^	—	—	—	—	1	0.633^∗∗^	−0.532^∗∗^	−0.550^∗∗^
PsyCap	122.62 ± 23.74	40.14 ± 5.43^∗∗^	—	—	—	—	—	1	−0.525^∗∗^	−0.564^∗∗^
PS	26.96 ± 6.25	38.74 ± 5.06^∗∗^	—	—	—	—	—	—	1	0.750^∗∗^
DS	20.68 ± 10.97	41.27 ± 6.72^∗∗^	—	—	—	—	—	—	—	1

*Note:* The normality parameters were derived from the results of the Kolmogorov–Smirnov test. Spearman’s rank correlation test was applied for the correlation analyses.

Abbreviations: Ag, agreeableness; C, conscientiousness; DS, depressive symptoms; E, extraversion; N, neuroticism; NP, normality parameters; O, openness; PsyCap, psychological capital; PS, perceived stress; PT, personality trait; SD, standard deviation.

^∗^ *p* < 0.01

^∗∗^ *p* < 0.001.

### 4.5. Regression Analysis

The detailed model estimates of the regression results for the five PT dimensions are presented in Tables S1–S5. After adjustment for covariates, neuroticism was positively associated with DS (*β* = 0.39, *p* < 0.001) and PS (*β* = 0.67, *p* < 0.001) and negatively associated with PsyCap (*β* = −0.57, *p* < 0.001). In contrast, extraversion, openness to experience, agreeableness, and conscientiousness demonstrated the opposite pattern, showing positive associations with PsyCap (*β* = 0.28–0.67, all *p* < 0.01) and negative associations with PS (*β* = −0.38 to −0.20, all *p* < 0.001). Among these four positive PT dimensions, only extraversion did not retain a statistically significant direct association with DS (*β* = −0.05, *p* > 0.05). Across all five regression models, PsyCap was negatively associated with both PS (*β* = −0.51 – −0.22, all *p* < 0.001) and DS (*β* = −0.15 to −0.09, all *p* < 0.01), whereas PS was positively associated with DS (*β* = 0.41–0.66, all *p* < 0.01). In addition, all VIF values were below 5 (maximum VIF = 2.962), indicating no evidence of problematic multicollinearity.

### 4.6. Mediating Effects Analysis

**Figure 2:**
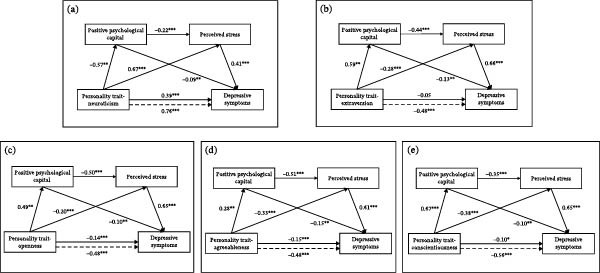
Multiple mediation models with standardized path coefficients. (a) The mediation model of neuroticism on depressive symptoms; (b) the mediation model of extraversion on depressive symptoms; (c) the mediation model of openness to experience on depressive symptoms; (d) the mediation model of agreeableness on depressive symptoms; (e) the mediation model of conscientiousness on depressive symptoms.

**Figure 3:**
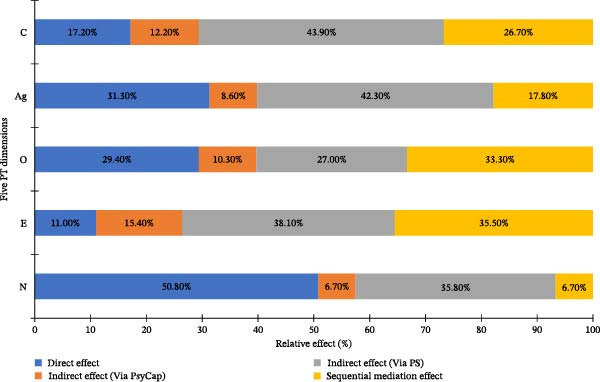
Comparison of the direct and indirect effects of the five personality trait dimensions on depressive symptoms. Abbreviations: Ag, agreeableness; C, conscientiousness; E, extraversion; N, neuroticism; O, openness to experience; PS, perceived stress; PsyCap, psychological capital; PT, personality trait.

Mediation analyses showed statistically significant indirect effects for all five PT dimensions through PsyCap, PS, or their proposed sequential pathway (Table 4 and Figure 2a–e). For neuroticism (Figure 2a), the standardized total effect on DS was 0.76, with a direct effect of 0.39 (50.8% of the total effect) and an indirect effect of 0.37 (49.2% of the total effect), indicating partial statistical mediation. The 95% bias‐corrected CIs for all three indirect pathways excluded zero, supporting the statistical significance of the indirect effects. Similar patterns of partial statistical mediation were observed for openness to experience, agreeableness, and conscientiousness (Figure 2c–e). In contrast, for extraversion (Figure 2b), the direct association with DS was no longer statistically significant after PsyCap and PS were included in the model, suggesting that its association with DS was primarily reflected through the indirect pathways. To facilitate comparisons across the five PT dimensions, Figure 3 presents the standardized direct effect and three indirect pathways (via PsyCap, via PS, and via PsyCap→PS) for each mediation model. The figure illustrates distinct effect patterns across PT dimensions. Specifically, neuroticism exhibited the largest direct association with DS, whereas the association between extraversion and DS was primarily reflected through indirect pathways. Openness to experience, agreeableness, and conscientiousness displayed similar effect patterns, with both direct and indirect pathways contributing to their associations with DS.

**Table 4:** Total, direct, and indirect effects of five personality trait dimensions on depressive symptoms (*N* = 583).

PTs	Effect type	Effect size	*β*	Bootstrap SE	Bootstrap 95% CI		Relative effect (%)
					LLCI	ULCI	
N	Total effect	1.15	0.76	0.04	1.07	1.23	100.0
	Direct effect	0.58	0.39	0.06	0.46	0.70	50.8
	Indirect effect	0.57	0.37	0.06	0.46	0.68	49.2
	N→PsyCap→DS	0.08	0.05	0.04	0.01	0.15	6.7
	N→PS→DS	0.41	0.27	0.05	0.32	0.51	35.8
	N→PsyCap→PS→DS	0.08	0.05	0.02	0.05	0.12	6.7
E	Total effect	−0.86	−0.48	0.06	−0.98	−0.72	100.0
	Direct effect	−0.09	−0.05	0.06	−0.21	0.02	11.0
	Indirect effect	−0.76	−0.43	0.06	−0.89	−0.64	89.0
	E→PsyCap→DS	−0.13	−0.07	0.05	−0.23	−0.04	15.4
	E→PS→DS	−0.33	−0.18	0.06	−0.44	−0.21	38.1
	E→PsyCap→PS→DS	−0.30	−0.17	0.04	−0.39	−0.23	35.5
O	Total effect	−0.97	−0.48	0.08	−1.11	−0.82	100.0
	Direct effect	−0.28	−0.14	0.06	−0.41	−0.16	29.4
	Indirect effect	−0.68	−0.34	0.07	−0.81	−0.55	70.6
	O→PsyCap→DS	−0.10	−0.05	0.05	−0.20	−0.01	10.3
	O→PS→DS	−0.26	−0.13	0.07	−0.40	−0.12	27.0
	O→PsyCap→PS→DS	−0.32	−0.16	0.04	−0.40	−0.24	33.3
Ag	Total effect	−1.04	−0.48	0.08	−1.20	−0.88	100.0
	Direct effect	−0.32	−0.15	0.06	−0.45	−0.20	31.3
	Indirect effect	−0.71	−0.33	0.06	−0.84	−0.59	68.7
	Ag→PsyCap→DS	−0.09	−0.04	0.03	−0.16	−0.03	8.6
	Ag→PS→DS	−0.44	−0.20	0.06	−0.56	−0.33	42.3
	Ag→PsyCap→PS→DS	−0.19	−0.09	0.03	−0.26	−0.12	17.8
C	Total effect	−0.92	−0.56	0.06	−1.03	−0.81	100.0
	Direct effect	−0.16	−0.10	0.06	−0.28	−0.04	17.2
	Indirect effect	−0.76	−0.47	0.07	−0.90	−0.63	82.8
	C→PsyCap→DS	−0.11	−0.07	0.05	−0.22	−0.004	12.2
	C→PS→DS	−0.40	−0.25	0.06	−0.54	−0.28	43.9
	C→PsyCap→PS→DS	−0.25	−0.15	0.04	−0.33	−0.17	26.7

Abbreviations: 95% CI, 95% confidence interval; Ag, agreeableness; C, conscientiousness; DS, depressive symptoms; E, extraversion; LLCI, lower limit of the 95% confidence interval; N, neuroticism; O, openness; PsyCap, psychological capital; PS, perceived stress; PTs, personality traits; ULCI, upper limit of the 95% confidence interval.

Overall, the mediation results were consistent with nearly all of the proposed hypotheses (Table 5). Among the five PT dimensions, neuroticism was positively associated with DS and PS and negatively associated with PsyCap, whereas extraversion, openness to experience, agreeableness, and conscientiousness demonstrated the opposite pattern. In addition, PsyCap was negatively associated with both PS and DS, whereas PS was positively associated with DS. Significant indirect effects were observed through PsyCap alone, PS alone, and the proposed sequential pathway through PsyCap and PS. Collectively, these findings were consistent with the proposed sequential mediation model, suggesting that the associations between the five PT dimensions and DS may be statistically explained, in part, through variations in PsyCap and PS.

**Table 5:** Results of hypothesis testing.

Hypothesis	Supported	Test result
H1a–e	H1a, c, d, e Yes H1b No	PT‐N positively influences DS; PT‐O, PT‐Ag, and PT‐C negatively influences DS. PT‐E did not exhibit a significant direct effect on DS.
H2a–e	All yes	PT‐N negatively influences PsyCap; PT‐E, PT‐O, PT‐Ag, and PT‐C positively influences PsyCap.
H3	All yes	PsyCap negatively influences DS.
H4a–e	All yes	PsyCap mediates the relationship between five PT dimensions and DS.
H5a–e	All yes	PT‐N positively influences PS; PT‐E, PT‐O, PT‐Ag, and PT‐C negatively influences PS.
H6	All yes	PS positively influences DS.
H7a–e	All yes	PS mediates the relationship between five PT dimensions and DS.
H8	All yes	PsyCap negatively influences PS.
H9a–e	All yes	Five PT dimensions influences DS through a chain mediation pathway involving PsyCap and PS.

Abbreviations: Ag, agreeableness; C, conscientiousness; DS, depressive symptoms; E, extraversion; N, neuroticism; O, openness; PsyCap, psychological capital; PS, perceived stress; PT, personality trait.

## 5. Discussion

The present study examined the associations between the five PT dimensions and DS among medical students, with particular attention to the proposed sequential mediation model involving PsyCap and PS. Guided by the FFM of personality, positive psychology, and the cognitive appraisal theory of stress, this study evaluated whether the associations between PT dimensions and DS were statistically explained through PsyCap and PS. Preliminary analyses indicated no substantial CMB and supported the reliability and validity of the measurement instruments. The findings showed that the five PT dimensions were significantly associated with DS either directly or indirectly, with the exception that the direct association between extraversion and DS was not statistically significant. In addition, PsyCap and PS each demonstrated significant independent indirect effects, and the proposed sequential mediation pathway was statistically significant across all five PT dimensions. Taken together, these findings are consistent with the proposed conceptual model and suggest that stable personality characteristics and modifiable psychological resources may jointly contribute to individual differences in DS among medical students. Given the cross‐sectional design, these findings should be interpreted as statistical associations rather than evidence of causal relationships.

### 5.1. Prevalence of DS Among Chinese Medical Students

Using a CES‐D cutoff score of ≥20, 60.9% of the medical students in the present study screened positive for clinically relevant DS. This prevalence appears to be higher than that reported in many pre‐pandemic studies of Chinese and international medical students [11, 124], although differences in assessment instruments, cutoff values, study populations, and sampling strategies should be considered when making direct comparisons. Nevertheless, this finding reinforces growing concerns regarding the mental health of medical students and underscores the importance of identifying the psychological factors associated with DS in this population. One plausible explanation for the relatively high prevalence observed in this study is the timing of data collection. The survey was conducted between November 2021 and January 2022, when strict COVID‐19 prevention and control measures were still implemented in China. Previous studies have shown that the COVID‐19 pandemic was associated with increased levels of depression and psychological distress among medical students through educational disruptions, reduced social interaction, uncertainty regarding academic progression, and concerns about future clinical training [23, 125]. Therefore, the relatively high prevalence observed in the present study should be interpreted within this specific historical context rather than generalized to medical students under routine educational conditions.

### 5.2. PTs and DS

The present study found that most PT dimensions were significantly associated with DS among medical students, although the direct association between extraversion and DS was not statistically significant after accounting for the proposed mediators. Overall, these findings are broadly consistent with the FFM of personality, which suggests that neuroticism is associated with increased vulnerability to depression, whereas openness to experience, agreeableness, and conscientiousness are generally associated with better psychological adjustment. Similar patterns have been reported in previous studies of medical students from different cultural backgrounds. For example, Tuygar Okutucu and Ceyhun [126] found that depression was positively associated with neuroticism and negatively associated with the remaining four PT dimensions among Turkish medical students. Likewise, Duica et al. [127] reported a positive association between neuroticism and depression among Romanian medical students. Pitanupong et al. [128] similarly observed that neuroticism was positively associated with depression, whereas extraversion, agreeableness, and conscientiousness were negatively associated with depression in Thai medical students; however, unlike the present study, they did not identify a significant association between openness to experience and depression. Taken together, these findings suggest that the associations between PT dimensions and DS are generally consistent across medical student populations, although some variation exists across different educational and cultural settings. Several factors may account for the minor inconsistencies observed across studies. First, cultural values and educational environments may influence both the expression of PTs and the psychological responses to academic stress. Medical students from different countries are exposed to diverse curricula, assessment systems, and clinical training environments, which may influence how PTs are expressed and how they relate to DS [129]. Second, differences in participant characteristics and study design may also contribute to heterogeneous findings. For example, the present study focused exclusively on third‐year clinical medicine undergraduates, whereas previous studies included students from multiple academic years.

From a theoretical perspective, several psychological processes may help explain the observed associations. Individuals high in neuroticism are generally more likely to exhibit heightened emotional reactivity, maladaptive emotion regulation strategies, and repetitive negative thinking (e.g., rumination), psychological processes that have been consistently associated with vulnerability to depression [130–132]. Conversely, agreeableness and conscientiousness are typically associated with stronger self‐regulation, greater persistence, and more adaptive interpersonal functioning, characteristics that facilitate effective coping with stressful life events [51, 133, 134]. Openness to experience has also been linked to greater cognitive flexibility, which may promote more adaptive responses to challenging situations [135]. These psychological characteristics provide plausible explanations for why different PT dimensions may show distinct associations with DS. In the present study, the absence of a statistically significant direct association between extraversion and DS suggests that its relationship with DS may be more strongly reflected through psychological resources and stress appraisal than through a direct association.

Furthermore, previous neurobiological research provides one possible perspective for understanding these behavioral findings. Studies have suggested that neuroticism and depression share partially overlapping genetic influences [136, 137] and that neuroticism is associated with altered activity and connectivity in brain regions involved in emotion regulation, including the amygdala, anterior cingulate cortex, and prefrontal cortex [138–140]. Conversely, agreeableness and conscientiousness have been associated with more effective prefrontal regulation of emotional responses [141]. Neuroimaging studies have also linked extraversion to reward‐processing networks involving the ventral striatum and orbitofrontal cortex, which may contribute to positive affect without necessarily showing a strong direct association with DS under conditions of sustained stress [142]. However, these neurobiological findings were not examined in the present study and should therefore be regarded as possible explanations derived from previous research rather than mechanisms established by the current findings. Overall, the present results suggest that PT dimensions may be associated with DS through multiple behavioral, psychological, and potentially neurobiological pathways, highlighting the importance of considering both direct and indirect associations when examining depression among medical students.

### 5.3. Mediating Effect of PsyCap

The present study found that PsyCap served as a significant statistical mediator in the association between PT dimensions and DS among medical students. This finding is consistent with the Broaden‐and‐build theory of positive emotions, which proposes that positive psychological resources broaden individuals’ cognitive and behavioral repertoires while facilitating the accumulation of enduring psychological resources for coping with adversity [38, 143–145]. From the perspective of positive psychology, mental health is influenced not only by vulnerability factors but also by the availability of positive psychological resources. Accordingly, individuals with higher levels of extraversion, openness to experience, agreeableness, and conscientiousness may be more likely to develop positive psychological resources, including hope, optimism, resilience, and self‐efficacy [65], whereas higher neuroticism may hinder the development of these resources through greater emotional instability and maladaptive cognitive styles [146]. The present findings are also consistent with previous studies reporting that PsyCap mediates the associations between a variety of psychosocial factors and DS among college and medical student populations [60, 76, 77]. The current findings extend this evidence by suggesting that PsyCap may also represent an important pathway through which PT dimensions are statistically associated with DS among medical students.

Several psychological mechanisms may help explain these associations. Individuals with greater PsyCap are generally more likely to maintain confidence when facing challenges, remain optimistic about future outcomes, persist toward goals despite setbacks, and recover more effectively from adversity [61]. These characteristics may facilitate adaptive coping, enhance motivation and problem‐solving, and reduce vulnerability to DS [147]. Conversely, lower PsyCap may limit individuals’ ability to mobilize psychological resources when confronted with academic and clinical stressors, thereby increasing susceptibility to DS [61]. These interpretations are consistent with positive psychology theory but were not directly examined in the present study. From an applied perspective, the present findings suggest that PsyCap may represent a promising target for promoting positive psychological development among medical students. Previous systematic reviews and meta‐analyses have shown that positive psychology interventions and strengths‐based educational programs can effectively enhance positive psychological resources and well‐being among student populations [148–152]. Accordingly, integrating these resource‐oriented approaches into medical education may help strengthen students’ PsyCap and improve their capacity to cope with future academic and professional challenges. Nevertheless, because the present study employed a cross‐sectional design, future longitudinal, and intervention studies are needed to determine whether enhancing PsyCap subsequently contributes to reductions in DS.

### 5.4. Mediating Effect of PS

PS was identified as an important statistical pathway linking PT dimensions and DS among medical students. Rather than merely reflecting exposure to stressful events, PS represents individuals’ subjective appraisal of environmental demands and their perceived ability to cope with them. This finding is consistent with the cognitive appraisal theory of stress, which emphasizes that psychological responses depend not only on objective stressors but also on how individuals interpret and evaluate those stressors [79]. Previous studies have reported both consistent and inconsistent findings regarding the relationships among PTs, PS, and DS. Sfeir et al. [153] reported that, among Lebanese medical students, higher neuroticism was associated with lower PS, whereas openness to experience and agreeableness were positively associated with PS, which differs from the present findings. Although both studies focused on medical students, the discrepant results may partly reflect differences in the sociocultural context, medical education systems, academic stressors, measurement approaches, and sample characteristics. In contrast, Kim et al. [87] using a large Korean health‐screening cohort, found that PS statistically mediated the associations between PTs and DS. However, because their sample consisted of adults undergoing health screening rather than medical students, direct comparison with the present findings should be made cautiously.

Several psychological explanations may account for the observed mediation pattern. Individuals with higher neuroticism may be more likely to interpret academic and clinical demands as threatening, engage in maladaptive cognitive styles such as rumination and catastrophizing, and perceive themselves as less capable of coping with stressors, thereby reporting higher PS and greater DS [132, 154]. Conversely, individuals with higher extraversion, openness to experience, agreeableness, and conscientiousness may be more likely to use adaptive coping strategies and social support, which may reduce subjective stress appraisal and lower vulnerability to DS [155]. Thus, PS may serve as a modifiable psychological process through which PT dimensions are statistically associated with DS. From an applied perspective, the present findings suggest that interventions targeting stress appraisal and stress management may be beneficial for medical students experiencing elevated levels of DS. Previous systematic reviews have shown that mindfulness‐based and other evidence‐based stress management interventions can effectively reduce PS and improve psychological adjustment among university and medical student populations [156–159]. Accordingly, integrating these approaches into medical education may help students respond more adaptively to academic and clinical demands. Nevertheless, because the present study employed a cross‐sectional design, future longitudinal and intervention studies are needed to determine whether interventions that reduce PS subsequently lead to improvements in DS.

### 5.5. Sequential Mediating Effects of PsyCap and PS

The present study found that the proposed sequential mediation model involving PsyCap and PS was statistically significant. Specifically, the associations between the five PT dimensions and DS were consistent with an indirect pathway through PsyCap and PS. Although the cross‐sectional design does not permit conclusions regarding temporal or causal relationships, these findings are consistent with the proposed conceptual model, in which personality dispositions are associated with psychological resources, which in turn are related to stress appraisal and DS.

The proposed sequential pathway was developed on the basis of both theoretical and empirical considerations. According to the Broaden‐and‐build theory of positive emotions and the transactional model of stress and coping, positive psychological resources broaden individuals’ cognitive and behavioral repertoires, enhance coping capacity, and influence how stressful situations are appraised rather than the objective occurrence of stressors themselves [160, 161]. Because PS reflects individuals’ subjective evaluation of environmental demands, PsyCap is theoretically expected to precede PS within the proposed framework. This conceptualization is also supported by previous empirical studies reporting that higher PsyCap is associated with lower levels of PS and better psychological adjustment [93]. Nevertheless, alternative pathways remain plausible, and future longitudinal or experimental studies are needed to determine the temporal ordering of PsyCap and PS more conclusively.

Although medical students are routinely exposed to substantial academic and personal stressors, the psychological impact of these experiences depends largely on how such stressors are perceived and appraised [79, 162]. Individuals with lower PsyCap may be more likely to interpret stressful situations negatively, resulting in higher levels of PS and greater vulnerability to DS [163, 164]. Conversely, prolonged exposure to elevated PS may gradually diminish positive psychological resources, suggesting that reciprocal relationships between PsyCap and PS are also possible [165]. Previous neurobiological studies have proposed several mechanisms that may help explain these associations, including more effective emotion regulation and attenuated physiological stress responses among individuals with greater psychological resources [166, 167]. However, these mechanisms were not examined in the present study and therefore should be regarded as potential explanations rather than direct evidence for the observed associations. Collectively, these findings suggest that PsyCap and PS should not be viewed as isolated mediators but as interrelated psychological processes through which PTs are associated with DS.

### 5.6. Theoretical and Practical Implications

The present study contributes to the literature on personality and depression among medical students by integrating PsyCap and PS into a single sequential mediation framework. Rather than viewing PTs solely as relatively stable predispositional factors, the proposed model suggests that their associations with DS may be partly explained through modifiable psychological resources and stress appraisal processes. In this way, the present findings extend traditional personality–psychopathology frameworks, such as the vulnerability–stress model [168], by highlighting the potential interplay between relatively stable personality characteristics and potentially modifiable psychological factors [169]. Although the cross‐sectional design precludes causal inference, the proposed framework provides a useful conceptual basis for future longitudinal and experimental research examining the psychological mechanisms linking personality and depression.

From a practical perspective, the present findings suggest that both PsyCap and PS may represent promising targets for mental health promotion among medical students. Existing intervention studies have shown that approaches such as resilience training, mindfulness‐based stress reduction, cognitive–behavioral interventions designed to enhance optimism and self‐efficacy, and programs that strengthen peer and faculty support can improve positive psychological resources and stress management [170–173]. Although the effectiveness of these interventions was not evaluated in the present study, integrating such evidence‐informed approaches into medical education may complement existing student mental health services. Future intervention studies are warranted to determine whether enhancing PsyCap and improving stress appraisal can ultimately reduce DS in medical students.

### 5.7. Limitations and Future Direction

Several limitations should be considered when interpreting the present findings. First, the cross‐sectional design precludes conclusions regarding temporal or causal relationships among PT, PsyCap, PS, and DS. Although the proposed sequential mediation model was theoretically informed, the observed indirect effects should be interpreted as statistical associations rather than evidence of causal pathways. Future longitudinal and experimental studies are needed to establish temporal ordering and examine the proposed mechanisms more rigorously.

Second, the study sample consisted exclusively of third‐year undergraduate clinical medicine students from three medical universities in Liaoning Province, China. Consequently, the findings may not be generalizable to medical students at different stages of training, students from other medical disciplines, practicing healthcare professionals, or populations from different cultural backgrounds. In addition, data collection was conducted during the COVID‐19 pandemic, which may have influenced both the prevalence of DS and the observed relationships among the study variables. Future multicenter and cross‐cultural studies are warranted to evaluate the generalizability of the proposed model under different educational and social contexts.

Third, all variables were assessed using self‐report questionnaires, which may be subject to reporting bias and CMB despite the procedural and statistical approaches adopted to minimize these concerns. Future studies could strengthen the evidence by incorporating multiple sources of information, including behavioral assessments, clinician ratings, or objective physiological indicators, where appropriate.

Finally, the present analyses were conducted using the PROCESS macro based on the observed variables. Although this approach is widely used for mediation analysis, it does not explicitly model latent constructs or account for measurement error. Future research could employ structural equation modeling or other latent variable approaches to provide a more comprehensive evaluation of the proposed conceptual framework. In addition, future studies may benefit from examining other potential mediators or moderators, such as social support, coping strategies, sleep quality, or academic burnout, to further clarify the psychological processes linking PTs and DS.

## 6. Conclusion

The present study examined the associations among PTs, PsyCap, PS, and DS in medical students using a sequential mediation framework. The findings suggest that most PT dimensions were associated with DS both directly and indirectly through PsyCap and PS, although the direct association between extraversion and DS was not statistically significant. These results highlight the potential importance of psychological resources and stress appraisal in understanding the associations between PTs and DS among medical students. Given the cross‐sectional design, the proposed mediation pathways should be interpreted as statistical associations rather than evidence of causal relationships. Future longitudinal, multi‐method, and intervention studies are needed to examine these pathways further and to determine whether strengthening PsyCap and improving stress appraisal can contribute to better mental health outcomes in medical students.

**Nomenclature**
95%CI: 95% confidence interval
AVE: average variance extracted
ANOVA: one‐way analysis of variance
CES‐D: The Center for Epidemiologic Studies Depression Scale
CMB: common method bias
COVID‐19: coronavirus disease 2019
CR: composite reliability
DS: depressive symptoms
EFA: exploratory factor analysis
FFM: five‐factor model
KMO: Kaiser–Meyer–Olkin
MOE: margin of error
NEO‐FFI‐R: The Neuroticism‐Extraversion‐Openness Five‐Factor Inventory‐Revised
P: prevalence
PCQ: The Psychological Capital Questionnaire
PPQ: The Positive Psychological‐capital Questionnaire
PS: perceived stress
PSS: The Perceived Stress Scale
PsyCap: psychological capital
PTs: personality traits
SD: standard deviation
VIF: variance inflation factor.

## Author Contributions

Ziqi Wang and Simeng Wang designed the study and collected data. Ziqi Wang analyzed data. Ziqi Wang drafted the manuscript. Simeng Wang and Deliang Wen contributed to the critical revision and approval of the final manuscript.

## Funding

This work was supported by the Liaoning Provincial Department of Science and Technology Doctoral Launch (Grant 2024‐BS‐056).

## Disclosure

All authors have read and approved the final manuscript.

## Ethics Statement

In accordance with the guidelines of ethical approval agencies and relevant Chinese laws and regulations, this study was reviewed and approved by the Ethics Committee under the Academic Committee of the China Medical University (approval number: 2019079). All participants voluntarily took part in the study and were fully informed of the research purpose, procedures, potential risks, and their rights. Participants who did not wish to complete the questionnaire could withdraw at any time without penalty, and no personal information would be recorded. The data collected were processed anonymously, used solely for academic purposes, and strictly adhered to confidentiality principles, ensuring no adverse effects on participants’ physical or psychological well‐being.

## Consent

The authors have nothing to report.

## Conflicts of Interest

The authors declare no conflicts of interest.

## Supporting Information

Additional supporting information can be found online in the Supporting Information section.

## Supporting information


**Supporting Information** Table S1: Regression analysis of the relationships between neuroticism and depressive symptoms. Table S2: Regression analysis of the relationships between extraversion and depressive symptoms. Table S3: Regression analysis of the relationships between openness and depressive symptoms. Table S4: Regression analysis of the relationships between agreeableness and depressive symptoms. Table S5: Regression analysis of the relationships between conscientiousness and depressive symptoms.

## Data Availability

The datasets used and/or analyzed during the current study are available from the corresponding author upon reasonable request.
