# Supplementary material for: Personality Trait Dimensions and Depressive Symptoms Among Medical Students: Sequential Mediation Through Psychological Capital and Perceived Stress
Source: Depress Anxiety. 2026 Aug 6;2026:9727081. doi: 10.1155/da/9727081 (PMC13444726; doi:10.1155/da/9727081)
Supplement: Supplementary file 1 [file DA-2026-9727081-s001.docx]

# Supplementary Materials

## Personality trait dimensions and depressive symptoms among medical students: Sequential mediation through psychological capital and perceived stress

## Contents

**Table S1. Regression analysis of the relationships between neuroticism and depressive symptoms**

**Table S2. Regression analysis of the relationships between extraversion and depressive symptoms**

**Table S3. Regression analysis of the relationships between openness and depressive symptoms**

**Table S4. Regression analysis of the relationships between agreeableness and depressive symptoms**

**Table S5. Regression analysis of the relationships between conscientiousness and depressive symptoms**

This Supplementary material has been provided by the authors to give readers additional information about their work. The publisher is not responsible for the content or functionality of any Supplementary material supplied by the authors. Any queries should be directed to the corresponding author for the article.

## Table S1. Regression analysis of the relationships between neuroticism and depressive symptoms (*N* = 583)

| **Predictor** | **Model 1 (DS)** | | | **Model 2 (PsyCap)** | | | **Model 3 (PS)** | | | **Model 4 (DS)** | | |
| --- | --- | --- | --- | --- | --- | --- | --- | --- | --- | --- | --- | --- |
|  | ***β*** | ***t*** | ***VIF*** | ***β*** | ***t*** | ***VIF*** | ***β*** | ***t*** | ***VIF*** | ***β*** | ***t*** | ***VIF*** |
| Constant |  | -5.09*** |  |  | 23.22*** |  |  | 7.21*** |  |  | -2.72** |  |
| Gender | -0.07 | -2.56* | 1.017 | -0.07 | -2.23* | 1.017 | 0.01 | 0.57 | 1.026 | -0.09 | -3.61*** | 1.026 |
| Place of residence | -0.01 | -0.27 | 1.807 | -0.02 | -0.45 | 1.807 | 0.01 | 0.40 | 1.808 | -0.02 | -0.58 | 1.808 |
| Only-child | -0.003 | -0.08 | 1.314 | 0.04 | 1.09 | 1.314 | -0.04 | -1.48 | 1.317 | 0.02 | 0.78 | 1.322 |
| Father’s education level | -0.04 | -1.00 | 2.194 | 0.06 | 1.11 | 2.194 | -0.03 | -0.83 | 2.198 | -0.02 | -0.49 | 2.201 |
| Mother’s education level | 0.04 | 1.04 | 2.442 | -0.02 | -0.42 | 2.442 | 0.01 | 0.26 | 2.443 | 0.04 | 0.94 | 2.443 |
| Exercise | 0.01 | 0.30 | 1.012 | 0.03 | 0.82 | 1.012 | 0.03 | 1.28 | 1.014 | 0.0002 | 0.01 | 1.016 |
| PTs-N | 0.76 | 28.02*** | 1.019 | -0.57 | -16.67*** | 1.019 | 0.67 | 22.34*** | 1.511 | 0.39 | 9.45*** | 2.826 |
| PsyCap |  |  |  |  |  |  | -0.22 | -7.35*** | 1.511 | -0.09 | -2.84** | 1.653 |
| PS |  |  |  |  |  |  |  |  |  | 0.41 | 9.78*** | 2.962 |
| *R* | 0.764 | | | 0.582 | | | 0.814 | | | 0.814 | | |
| *R²* | 0.584 | | | 0.338 | | | 0.662 | | | 0.662 | | |
| *F* | 115.45*** | | | 41.98*** | | | 140.80*** | | | 124.59*** | | |

**Note:** **p* < 0.05; ***p* < 0.01; ****p* < 0.001.

**Abbreviation:** DS, depressive symptoms; PsyCap, psychological capital; PS, perceived stress; PT-N, personality trait-neuroticism.

## Table S2. Regression analysis of the relationships between extraversion and depressive symptoms (*N* = 583)

| **Predictor** | **Model 1 (DS)** | | | **Model 2 (PsyCap)** | | | **Model 3 (PS)** | | | **Model 4 (DS)** | | |
| --- | --- | --- | --- | --- | --- | --- | --- | --- | --- | --- | --- | --- |
|  | ***β*** | ***t*** | ***VIF*** | ***β*** | ***t*** | ***VIF*** | ***β*** | ***t*** | ***VIF*** | ***β*** | ***t*** | ***VIF*** |
| Constant |  | 13.86*** |  |  | 4.73*** |  |  | 25.11*** |  |  | 0.68 |  |
| Gender | -0.09 | -2.47* | 1.016 | -0.06 | -1.87 | 1.016 | -0.02 | -0.56 | 1.022 | -0.10 | -3.96*** | 1.022 |
| Place of residence | 0.04 | 0.76 | 1.801 | -0.05 | -1.04 | 1.801 | 0.04 | 1.04 | 1.805 | -0.01 | -0.34 | 1.808 |
| Only-child | -0.01 | -0.30 | 1.316 | 0.03 | 0.91 | 1.316 | -0.04 | -1.20 | 1.317 | 0.03 | 1.03 | 1.321 |
| Father’s education level | 0.002 | 0.03 | 2.204 | 0.002 | 0.03 | 2.204 | 0.01 | 0.11 | 2.204 | -0.001 | -0.02 | 2.204 |
| Mother’s education level | 0.002 | 0.04 | 2.439 | 0.01 | 0.19 | 2.439 | -0.02 | -0.49 | 2.439 | 0.02 | 0.55 | 2.440 |
| Exercise | -0.003 | -0.08 | 1.015 | 0.02 | 0.52 | 1.015 | 0.02 | 0.75 | 1.015 | -0.01 | -0.44 | 1.016 |
| PT-E | -0.48 | -13.17*** | 1.019 | 0.59 | 17.63*** | 1.019 | -0.28 | -6.93*** | 1.570 | -0.05 | -1.56 | 1.701 |
| PsyCap |  |  |  |  |  |  | -0.44 | -10.91*** | 1.569 | -0.13 | -3.49*** | 1.895 |
| PS |  |  |  |  |  |  |  |  |  | 0.66 | 19.44*** | 1.717 |
| *R* | 0.495 | | | 0.602 | | | 0.646 | | | 0.782 | | |
| *R²* | 0.245 | | | 0.363 | | | 0.418 | | | 0.611 | | |
| *F* | 26.612*** | | | 46.747*** | | | 51.445*** | | | 99.879*** | | |

Note: **p* < 0.05; ***p* < 0.01; ****p* < 0.001.

**Abbreviation:** DS, depressive symptoms; PsyCap, psychological capital; PS, perceived stress; PT-E, personality trait-extraversion.

## Table S3. Regression analysis of the relationships between openness and depressive symptoms (*N* = 583)

| **Predictor** | **Model 1 (DS)** | | | **Model 2 (PsyCap)** | | | **Model 3 (PS)** | | | **Model 4 (DS)** | | |
| --- | --- | --- | --- | --- | --- | --- | --- | --- | --- | --- | --- | --- |
|  | ***β*** | ***t*** | ***VIF*** | ***β*** | ***t*** | ***VIF*** | ***β*** | ***t*** | ***VIF*** | ***β*** | ***t*** | ***VIF*** |
| Constant |  | 14.21*** |  |  | 3.90*** |  |  | 23.44*** |  |  | 2.24* |  |
| Gender | -0.07 | -1.77 | 1.020 | -0.09 | -2.41* | 1.020 | -0.01 | -0.37 | 1.030 | -0.09 | -3.63*** | 1.030 |
| Place of residence | -0.01 | -0.16 | 1.818 | -0.005 | -0.10 | 1.818 | 0.02 | 0.55 | 1.818 | -0.03 | -0.73 | 1.819 |
| Only-child | 0.01 | 0.21 | 1.324 | 0.02 | 0.46 | 1.324 | -0.03 | -0.93 | 1.325 | 0.04 | 1.33 | 1.327 |
| Father’s education level | -0.002 | -0.03 | 2.203 | 0.01 | 0.28 | 2.203 | 0.0003 | 0.01 | 2.203 | 0.005 | 0.12 | 2.203 |
| Mother’s education level | 0.01 | 0.26 | 2.440 | -0.004 | -0.06 | 2.440 | -0.02 | -0.37 | 2.440 | 0.03 | 0.63 | 2.440 |
| Exercise | -0.03 | -0.74 | 1.009 | 0.05 | 1.38 | 1.009 | 0.01 | 0.38 | 1.012 | -0.01 | -0.53 | 1.013 |
| PT-O | -0.48 | -12.83*** | 1.044 | 0.49 | 13.25*** | 1.044 | -0.20 | -5.29*** | 1.363 | -0.14 | -4.58*** | 1.429 |
| PsyCap |  |  |  |  |  |  | -0.50 | -13.49*** | 1.330 | -0.10 | -2.95** | 1.751 |
| PS |  |  |  |  |  |  |  |  |  | 0.65 | 19.53*** | 1.662 |
| *R* | 0.485 | | | 0.498 | | | 0.631 | | | 0.789 | | |
| *R²* | 0.236 | | | 0.248 | | | 0.398 | | | 0.623 | | |
| *F* | 25.326*** | | | 27.072*** | | | 47.473*** | | | 105.162*** | | |

Note: **p* < 0.05; ***p* < 0.01; ****p* < 0.001.

**Abbreviation:** DS, depressive symptoms; PsyCap, psychological capital; PS, perceived stress; PT-O, personality trait-openness.

## Table S4. Regression analysis of the relationships between agreeableness and depressive symptoms (*N* = 583)

| **Predictor** | **Model 1 (DS)** | | | **Model 2 (PsyCap)** | | | **Model 3 (PS)** | | | **Model 4 (DS)** | | |
| --- | --- | --- | --- | --- | --- | --- | --- | --- | --- | --- | --- | --- |
|  | ***β*** | ***t*** | ***VIF*** | ***β*** | ***t*** | ***VIF*** | ***β*** | ***t*** | ***VIF*** | ***β*** | ***t*** | ***VIF*** |
| Constant |  | 14.46*** |  |  | 6.47*** |  |  | 26.90*** |  |  | 3.22** |  |
| Gender | -0.03 | -0.91 | 1.032 | -0.09 | -2.30* | 1.032 | 0.02 | 0.57 | 1.042 | -0.09 | -3.33*** | 1.042 |
| Place of residence | 0.01 | 0.20 | 1.809 | -0.04 | -0.81 | 1.809 | 0.02 | 0.45 | 1.811 | -0.02 | -0.61 | 1.812 |
| Only-child | -0.01 | -0.14 | 1.318 | 0.05 | 1.13 | 1.318 | -0.03 | -0.85 | 1.321 | 0.04 | 1.22 | 1.322 |
| Father’s education level | -0.02 | -0.38 | 2.196 | 0.05 | 0.78 | 2.196 | -0.0004 | -0.01 | 2.199 | 0.001 | 0.01 | 2.199 |
| Mother’s education level | -0.02 | -0.37 | 2.442 | 0.02 | 0.36 | 2.442 | -0.04 | -0.85 | 2.442 | 0.01 | 0.34 | 2.445 |
| Exercise | -0.05 | -1.23 | 1.008 | 0.07 | 1.69 | 1.008 | 0.004 | 0.14 | 1.013 | -0.02 | -0.65 | 1.013 |
| PT-Ag | -0.48 | -12.99*** | 1.029 | 0.28 | 6.87*** | 1.029 | -0.33 | -10.38 | 1.113 | -0.15 | -5.10*** | 1.322 |
| PsyCap |  |  |  |  |  |  | -0.51 | -15.90 | 1.102 | -0.15 | -4.63*** | 1.588 |
| PS |  |  |  |  |  |  |  |  |  | 0.61 | 17.34*** | 1.882 |
| *R* | 0.490 | | | 0.305 | | | 0.684 | | | 0.791 | | |
| *R²* | 0.240 | | | 0.093 | | | 0.469 | | | 0.626 | | |
| *F* | 25.914*** | | | 8.402*** | | | 63.251*** | | | 106.589*** | | |

Note: **p* < 0.05; ***p* < 0.01; ****p* < 0.001.

**Abbreviation:** DS, depressive symptoms; PsyCap, psychological capital; PS, perceived stress; PT-Ag, personality trait-agreeableness.

## Table S5. Regression analysis of the relationships between conscientiousness and depressive symptoms (*N* = 583)

| **Predictor** | **Model 1 (DS)** | | | **Model 2 (PsyCap)** | | | **Model 3 (PS)** | | | **Model 4 (DS)** | | |
| --- | --- | --- | --- | --- | --- | --- | --- | --- | --- | --- | --- | --- |
|  | ***β*** | ***t*** | ***VIF*** | ***β*** | ***t*** | ***VIF*** | ***β*** | ***t*** | ***VIF*** | ***β*** | ***t*** | ***VIF*** |
| Constant |  | 15.70*** |  |  | 4.36*** |  |  | 26.35*** |  |  | 1.15 |  |
| Gender | -0.07 | -1.99* | 1.018 | -0.09 | -2.83** | 1.018 | 0.002 | 0.06 | 1.032 | -0.10 | -3.75*** | 1.032 |
| Place of residence | 0.01 | 0.14 | 1.807 | -0.01 | -0.27 | 1.807 | 0.03 | 0.67 | 1.808 | -0.02 | -0.44 | 1.809 |
| Only-child | 0.01 | 0.22 | 1.320 | 0.01 | 0.30 | 1.320 | -0.03 | -0.91 | 1.321 | 0.03 | 1.11 | 1.323 |
| Father’s education level | -0.01 | -0.27 | 2.197 | 0.02 | 0.48 | 2.197 | -0.01 | -0.13 | 2.198 | -0.002 | -0.07 | 2.198 |
| Mother’s education level | 0.05 | 0.85 | 2.445 | -0.04 | -0.87 | 2.445 | 0.004 | 0.09 | 2.448 | 0.03 | 0.72 | 2.448 |
| Exercise | 0.01 | 0.30 | 1.017 | 0.003 | 0.10 | 1.017 | 0.03 | 1.00 | 1.017 | -0.01 | -0.34 | 1.019 |
| PT-C | -0.56 | -16.13*** | 1.040 | 0.67 | 21.29*** | 1.040 | -0.38 | -9.05*** | 1.859 | -0.10 | -2.56* | 2.124 |
| PsyCap |  |  |  |  |  |  | -0.35 | -8.30*** | 1.822 | -0.10 | -2.77** | 2.041 |
| PS |  |  |  |  |  |  |  |  |  | 0.65 | 18.52*** | 1.810 |
| *R* | 0.569 | | | 0.672 | | | 0.669 | | | 0.783 | | |
| *R²* | 0.323 | | | 0.451 | | | 0.448 | | | 0.613 | | |
| *F* | 39.232*** | | | 67.496*** | | | 58.146*** | | | 101.052*** | | |

Note: **p* < 0.05; ***p* < 0.01; ****p* < 0.001.

**Abbreviation:** DS, depressive symptoms; PsyCap, psychological capital; PS, perceived stress; PT-C, personality trait-conscientiousness.
